# Supplementary material for: Drug repurposing for aging research using model organisms
Source: Aging Cell. 2017 Jun 16;16(5):1006–15. doi: 10.1111/acel.12626 (PMC5595691; doi:10.1111/acel.12626)
Supplement: Supplementary file 7 — Data S1 Zip‐Archive of all report cards. [file ACEL-16-1006-s007.zip › RC_PKA.pdf]

PKA

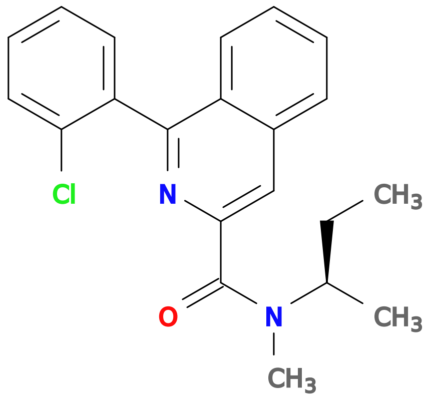

Database identifiers

ChEMBLCompound	CHEMBL416715
ZINC	ZINC03870481

Ranking

	Rank	Score
Drosophila	309/697	0.517
C. elegans	415/591	0.128

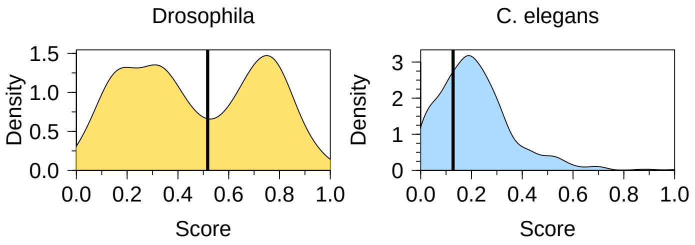

	Ageing implication	Domain conservation	Binding site conservation	Binding affinity	Bioavailability	Lipinski	Promiscuity	Purchasability	Drug approval	**Total**
Drosophila	0.792	0.906	0.767	0.944	(0.9)	-0.05	-0.0	0.1	0.0	0.517
C. elegans	0.792	0.796	0.576	0.944	0.228	-0.05	-0.0	0.1	0.0	0.128

Names

· $293393_aldrich4-02-00-00929(BeilsteinHandbookReference)$
· 6-Aminohexanoic acid homopolymer
· A 1030n0
· Akulon
· Alkamid
· Amilan CM 1031
· ATM 2 (nylon)
· Aviamide-6
· Capran 77C
· Capran 80
· Caproamide
· Caprolactam oligomer

· Caprolactam polymer
· Caprolon B
· Capron 8252
· Capron GR 8256
· Capron PK 4
· Ccris 4822
· Chemlon
· Danamid
· Durethan BK
· Durethan BKV 55H
· Einecs 211-024-8
· epsilon-Caprolactam polymere German
· Ertalon 6SA
· Extron 6N
· Grilon
· Hexahydro-2H-azepin-2-one homopolymer
· Hexamide
· Itamid
· Kaprolit B
· Kapron
· Ks 30p
· Maranyl F 124
· Maranyl F 500
· Miramid H 2
· Miramid WM 55
· Nci-c02142
· Nylon
· Orgamid rmnocd
· Orgamide
· P 6 (Polyamide)
· PA 6 (Polymer)
· Plaskin 8200
· Plaskon XP 607
· Policapram USAN:INN
· Policapramum INN-Latin
· Policapran
· Poly(epsilon-aminocaproic acid)
· Poly(hexahydro-2H-azepin-2-one)
· Poly(imino(1-oxo-1,6 hexanediyl))
· Poly(iminocarbonylpentamethylene)
· Polyamide 6
· Polycaproamide
· Polycaprolactam
· Relon P
· Renyl MV
· Sipas 60
· Spencer 401
· Tarlon X-A

· Tarnamid T
· Tnk 2g5
· Torayca N 6
· Ube 1022b
· Ultramid B 3
· Vidlon
· Zytel 211

Roles

ChEBI entry None has no roles

Status

Approved drug (according to ChEMBL)	No
Number of Rule of 5 violations	1
Binding affinity to original target in log units (RF-Score prediction)	7.82
Burns *C. elegans* bioavailability prediction	-3.46

Compound Target Characteristics

Translocator protein

Best gene implication in ageing for this target family came from gene P16257 annotated in UniProt release 2014_02. Annotation GO 7568 (aging) was Inferred from Expression Pattern

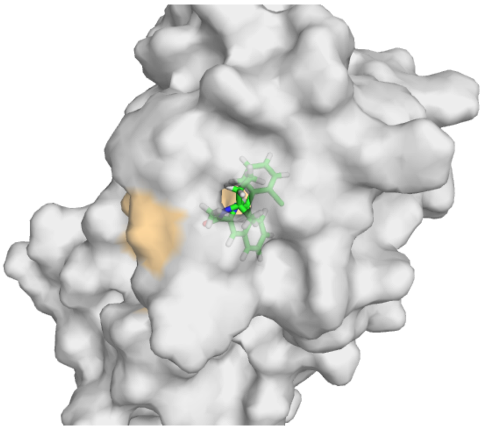

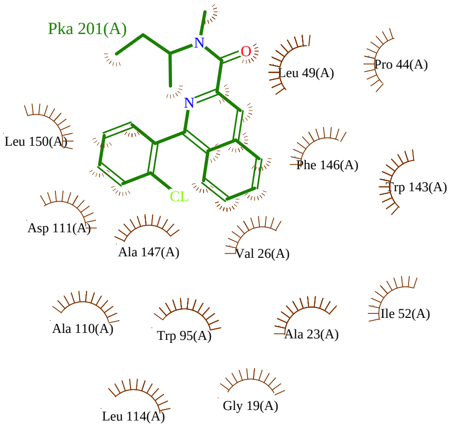

protein	amino acids contacts (binding site)
PDB:2mgy:chainA:P50637	G A V P L I W A D L W F A L
sp:P30536:TSPOA_HUMAN	C S V P L V W V D L W F T L
sp:P16257:TSPO_RAT	G A V P L I W V D L W F A L
sp:P50637:TSPO_MOUSE	G A V P L I W A D L W F A L
tr:Q541E3:Q541E3_MOUSE	G A V P L I W A D L W F A L
tr:Q9VPR1:Q9VPR1_DROME	G G T P F M W I D A W F A L
tr:B3WFW9:B3WFW9_CAEEL	A S K P Y V T K N V W F Y L
tr:C7FZU5:C7FZU5_CAEEL	A S K P Y V T K N V W F Y L

protein	whole protein ident	simil	domain-based ident	simil	contact-based ident	simil
PDB:2mgy:chainA:P50637	0.99	1.0	0.99	1.0	1.0	1.0
sp:P30536:TSPOA_HUMAN	0.81	0.94	0.83	0.95	0.64	0.73
sp:P16257:TSPO_RAT	0.95	0.99	0.97	0.99	0.93	0.96
sp:P50637:TSPO_MOUSE	1.0	1.0	1.0	1.0	1.0	1.0
tr:Q541E3:Q541E3_MOUSE	1.0	1.0	1.0	1.0	1.0	1.0
tr:Q9VPR1:Q9VPR1_DROME	0.38	0.75	0.46	0.83	0.57	0.77
tr:B3WFW9:B3WFW9_CAEEL	0.25	0.68	0.28	0.74	0.29	0.58
tr:C7FZU5:C7FZU5_CAEEL	0.21	0.56	0.28	0.74	0.29	0.58

CG2789 (FBgn0031263) associated phenotypes

chemical resistant, conditional, decreased cell death, long lived, male limited, oxidative stress response defective, radiation resistant

(Information from FlyBase)
